# Neural correlates of facial recognition deficits in autism spectrum disorder: a comprehensive review

**DOI:** 10.3389/fpsyt.2024.1464142

**Published:** 2025-01-06

**Authors:** Jianmei Liu, Huihui Chen, Haijing Wang, Zhidan Wang

**Affiliations:** ^1^ School of Public Policy and Management, China University of Mining and Technology, Xuzhou, China; ^2^ School of Education Science, Jiangsu Normal University, Xuzhou, China

**Keywords:** ASD, facial recognition deficits, FG, amygdala, STS, PFC, social cognition

## Abstract

Autism spectrum disorder (ASD) is a neurodevelopmental condition characterized by significant impairments in social interaction, often manifested in facial recognition deficits. These deficits hinder individuals with ASD from recognizing facial identities and interpreting emotions, further complicating social communication. This review explores the neural mechanisms underlying these deficits, focusing on both functional anomalies and anatomical differences in key brain regions such as the fusiform gyrus (FG), amygdala, superior temporal sulcus (STS), and prefrontal cortex (PFC). It has been found that the reduced activation in the FG and atypical activation of the amygdala and STS contribute to difficulties in processing facial cues, while increased reliance on the PFC for facial recognition tasks imposes a cognitive load. Additionally, disrupted functional and structural connectivity between these regions further exacerbates facial recognition challenges. Future research should emphasize longitudinal, multimodal neuroimaging approaches to better understand developmental trajectories and design personalized interventions, leveraging AI and machine learning to optimize therapeutic outcomes for individuals with ASD.

## 1 Introduction

Autism spectrum disorder (ASD) is a neurodevelopmental disorder that frequently becomes apparent during the formative years of childhood, characterized by substantial impairments in social interaction. These deficits are most commonly expressed through difficulties in communication, circumscribed interests, and the exhibition of repetitive or stereotypical behaviors (1). The World Health Organization has estimated that ASD impacts approximately one child in every hundred worldwide (2). The most recent data provided by the Centers for Disease Control and Prevention (CDC) indicate that the prevalence of ASD in the United States is as high as 2.76% and about 1 in 38 children aged 8 years has been diagnosed with ASD (3).

Research indicates that individuals with ASD often exhibit impairments in facial recognition and employ atypical strategies for facial recognition (4). Behavioral studies have shown that children with ASD are less likely to make eye contact and show reduced interest in faces compared to typically developing children (5–7). These deficits extend to difficulties in recognizing facial identities, interpreting facial expressions, and understanding social cues conveyed through facial movements (8). Recently, a review of the last four decades of empirical research on facial recognition in ASD has revealed that individuals with ASD perform, on average, one standard deviation below their neurotypical peers on tasks involving facial identity and discrimination (9). In addition, research has also highlighted atypical strategies used by individuals with ASD for facial recognition. For example, they may focus on non-facial features or use alternative cognitive processes to recognize faces, which can further hinder their social interactions (10). These strategies might involve concentrating on individual facial features rather than integrating them into a holistic representation, leading to less accurate and slower face recognition (11).

Given the importance of facial recognition in social interactions, these deficits may contribute significantly to the social challenges faced by individuals with ASD. For example, failing to recognize a partner’s distress or joy can lead to responses that seem insensitive or out of place, undermining social bonds (12). Additionally, atypical gaze behavior, such as focusing more on the mouth than the eyes during social interactions, has been found in individuals with ASD and is associated with decreased social competence (6). This altered gaze pattern suggests a reduced salience of the eyes, a critical component of non-verbal communication, which may contribute to difficulties in interpreting social cues and hinder effective social interactions. While previous studies have examined the neural mechanisms behind facial recognition deficits in individuals with ASD, a critical gap remains in understanding how key brain regions dynamically interact during real-time social interactions. Much of the existing research focuses on isolated regions such as the fusiform gyrus (FG) and amygdala, often neglecting how these regions communicate within larger networks, especially under different cognitive loads and social contexts. This review goes beyond individual activation levels to investigate both structural and functional connectivity between critical regions, such as the superior temporal sulcus and prefrontal cortex. By exploring these network dynamics and the compensatory mechanisms individuals with ASD may rely on, this review offers a more holistic model that better captures the complexity of social cognition in real-world scenarios.

In this review, we conducted a comprehensive search of articles on facial recognition deficits in individuals with ASD using multiple academic databases and platforms. The databases included PubMed, ScienceDirect, Web of Science, SpringerLink, Elsevier, as well as psycINFO, Scopus, and Google Scholar etc. We used a combination of search terms such as “autism,” “abnormal” “face recognition,” “dysfunctional face processing,” “autism face processing,” “autism face recognition,” “brain regions,” “neural network,” “brain connectivity,” “anatomical anomalies,” “autism face processing fMRI/MEG/fNIRS,” “AI,” and “machine learning.” We applied inclusion criteria to select studies that specifically investigated the neural mechanisms underlying facial recognition deficits in ASD, focusing on structural and functional brain anomalies, connectivity, and neural networks. Studies were excluded if they lacked a direct examination of facial recognition processes or did not involve neuroimaging or brain-based analyses. After applying the inclusion and exclusion criteria, we ultimately identified 70 relevant articles. Our aim was to synthesize these findings to construct a more systematic theoretical model that better explains the pathological mechanisms of facial recognition deficits in ASD.

## 2 Neural anomalies in brain regions of facial recognition in ASD

The brain structure of the core facial recognition network primarily encompasses the inferior occipital gyri (IOG), FG, superior temporal sulcus (STS), amygdala, and prefrontal cortex (PFC). These structures are tasked with the processing of both variable and invariant facial features (13). Based on the comprehensive literature review, we found that the facial recognition impairments observed in individuals with ASD are associated with underlying anatomical irregularities within this core facial network, including reduced activation in the FG, atypical amygdala activation, hypoactivity in the STS, and increased activation of the PFC.

### 2.1 Reduced activation in the FG

Research has consistently identified the FG as a key region associated with the recognition of human faces (14). This area is selectively activated during the processing of facial stimuli, leading to its popular designation as the “fusiform face area” (FFA) (15, 16). A comprehensive meta-analysis of 100 functional magnetic resonance imaging (fMRI) studies involving facial emotion recognition in typically developing individuals has underscored the pivotal role of the FG in facial recognition processes (17). Three discrete facial selective regions are localized within the middle fusiform sulci, the posterior fusiform gyrus, and the inferior occipital gyrus (18).

Some studies have revealed that individuals with ASD exhibit reduced activation in the FG during facial recognition tasks. Nickl-Jockschat et al. (19) conducted a meta-analysis on face processing in ASD, finding reduced activation in the FG. Functional connectivity analyses revealed that this region is connected to the temporo-occipital cortex, inferior frontal and parietal cortices, thalamus, and amygdala. These results suggest that disrupted face processing in ASD is part of a broader network related to face, affective, and language processing, potentially contributing to the observed impairments and reduced interest in faces. A study by Ibrahim (20) using advanced imaging techniques found persistent reductions in FG activation in children with ASD, reinforcing the link between structural anomalies and face processing deficits.

Furthermore, research has indicated that individuals with ASD may have a thinner cortex in the left FG compared to typically developing individuals, with age showing a significant negative correlation with cortical thickness in those with ASD, a relationship that is not observed in typically developing individuals (21). A stereotactic-based study offers a more nuanced neuroanatomical explanation for the structural changes within the fusiform gyrus, reporting a decreased neuronal density in layer III, a reduced total number of neurons across layers III, V, and VI, and a diminished mean perinuclear volume of neurons in layers V and VI of individuals with ASD (22).

### 2.2 Atypical amygdala activation

Research indicates that the amygdala is capable of processing significant amounts of sophisticated sensory information. Each amygdala neuron can respond to somatosensory, visual, auditory, and all types of visceral inputs. The afferent nerve carrying this information reaches the amygdala in the opposite direction along the path followed by the efferent nerve of the amygdala (23). A neuroimaging study has demonstrated that the human amygdala becomes active during the interpretation of social signals, including gaze, facial expression recognition, and body language (24). Nickl-Jockschat and colleagues (19) discovered that the amygdala is associated with emotional domains such as fear, disgust, happiness, and sadness through functional decoding.

Studies have revealed that individuals with ASD exhibit atypical amygdala activation across a range of tasks involving social cognition, including inferring mental states from pictures of eyes and evaluating facial expressions (25, 26). A fMRI study conducted by Baron-Cohen and his team (24) found that individuals with ASD had a markedly reduced activation of the amygdala during mentalizing tasks—such as inferring intentions from eyes or facial expressions—compared to typically developing individuals. In this study, researchers compared the activation status of functional systems between individuals with ASD (ASD group) and typically developing individuals (control group) through a series of tasks designed to assess the theory of mind. It was observed that the ASD group failed to engage the amygdala and exhibited less partial activation in the frontal lobe compared to the control group. Additionally, the control group demonstrated significantly enhanced response capabilities in the left amygdala, the right insula, and the left inferior FG, whereas the ASD group showed a significantly stronger response in the bilateral superior temporal gyrus. This suggests that rather than using the amygdala for information-processing tasks, individuals with ASD may shift the processing load to temporal lobe structures that are specialized for labeling complex visual stimuli, which could be a compensatory mechanism for the atypical functioning of the amygdala (24). Furthermore, Nordahl et al. (20) supports these findings, indicating that atypical amygdala activation patterns in ASD may begin early in development and persist into adolescence, and the altered amygdala activation in response to different social traits, such as trustworthiness and dominance, in individuals with ASD.

However, it is crucial to note that hypersensitive responses of the amygdala to facial stimuli in ASD have also been reported. Lassalle et al. (27) demonstrated that when gaze is controlled and constrained to the eyes, individuals with ASD show increased activation in social brain regions, including the amygdala, particularly in response to low-intensity fearful faces. This heightened sensitivity is coupled with a lack of functional correlation between the amygdala and the ventromedial prefrontal cortex, indicating a potential excitatory/inhibitory imbalance in socio-affective processing. These findings highlight the context-dependent nature of amygdala responses in ASD, suggesting that both hypoactivation and hypersensitivity may contribute to the atypical processing of social stimuli.

### 2.3 Hypoactivity in the STS

The STS is a critical region involved in social perception, particularly in processing eye gaze and interpreting the intentions and emotions conveyed through facial movements. Eye gaze serves as a critical social cue, and anomalies in gaze behavior are a notable characteristic among individuals with ASD. Research has consistently shown that individuals with ASD exhibit deficits in visual reciprocity and social gaze behavior, which are key indicators of facial recognition deficits in this population (7, 8). Direct gaze, where one individual looks directly into the eyes of another, has a task-relevant perceptual advantage over avoidance gaze (where gaze is directed away). Direct gaze is more conducive to categorization, recognition, and memory of facial information (28). In terms of neural activity, direct gaze requires more activation of the STS than avoidance gaze. This is because the STS is closely related to the perception and regulation of gaze behavior and is involved in decoding communicative intentions behind eye movements and processing the social meaning of movement cues (29, 30).

Several neuroimaging studies using dynamic facial stimulation have demonstrated the critical role of the STS in processing gaze direction. For example, research by Pelphrey et al. (7) showed that the STS responds selectively to direct versus averted gaze, suggesting that it plays a crucial role in understanding where others are looking and inferring their intentions. However, in individuals with ASD, these studies have failed to demonstrate the same level of STS activation and modulation by gaze direction (31–33). This finding aligns with results from Castelli et al. (34), which revealed hypoactivity in the STS and reduced functional connectivity between the STS and a portion of the inferior occipital gyrus (visual area V3) during tasks involving the attribution of intention to moving geometric shapes. These hypoactivity in the STS may contribute to the significant impairment in social gaze observed in individuals with ASD. The inability to effectively process and respond to direct gaze can hinder social interactions, as gaze behavior is integral to understanding and engaging in social communication.

Moreover, the STS is involved in the perception of biological motion and the integration of visual and auditory information to comprehend social actions and intentions (35). Fry et al. (31) identified diminished face-selective network connectivity and reduced STS selectivity in individuals with ASD. In individuals with ASD, the STS’s difficulty in extracting social cues from visual facial information may stem from underactivation of the core nodes within the somatosensory cortex network, which processes visual facial information. This underactivation results in reduced inputs to the mirror neuron system (MNS), further complicating the decoding of social signals (36). Research has shown that the STS’s role in social cognition extends beyond gaze processing. It is also implicated in understanding and predicting others’ actions and intentions, a process that requires integrating various social cues (29, 37). Studies using tasks that involve interpreting social interactions or attributing mental states to others have consistently found reduced STS activation in individuals with ASD, highlighting the broad impact of STS hypoactivity on social cognition (34).

### 2.4 The increased activation of PFC

The PFC is a crucial brain region involved in higher-order cognitive processes, including decision-making, social behavior, and emotional regulation. It plays a significant role in supporting complex cognitive functions such as planning, problem-solving, and regulating emotions (38). In individuals with ASD, atypical activation patterns in the PFC have been observed during tasks involving social cognition (39). Another study has provided evidence that individuals with ASD may recruit the PFC as a compensatory mechanism for deficits in the primary facial recognition network. Typically, face recognition relies heavily on the FFA, amygdala, and STS. However, due to anomalies in these regions, individuals with ASD might rely more on cognitive strategies mediated by the PFC to recognize faces and interpret social cues (40). To support this, Kim et al. (41) demonstrated that verbalization tasks improved facial recognition in adolescents with ASD by engaging the PFC. This compensatory strategy may help mitigate deficits in the fusiform gyrus and amygdala, which are commonly underactivated in individuals with ASD.

The prefrontal cortex is known for its role in executive functions, which include managing attention, regulating behavior, and controlling impulses (38). These functions are essential for adaptive social behavior and emotional regulation. In neurotypical individuals, the automatic processing of social information is primarily mediated by the FFA, amygdala, and STS. However, the reliance on the PFC suggests that individuals with ASD may engage more in conscious, effortful processing of social information (42). For example, a study by Gilbert et al. (39) found that during tasks requiring social cognition, individuals with ASD showed greater activation in the medial prefrontal cortex (mPFC) compared to neurotypical controls. This increased activation was interpreted as evidence of compensatory recruitment of the PFC to support social processing. Similarly, Lombardo et al. (40) observed that individuals with ASD exhibited atypical patterns of activation in the PFC when interpreting social cues, suggesting that they might be compensating for deficits in more intuitive social processing networks.

This compensatory strategy, while potentially helpful in addressing some deficits, can be cognitively demanding. The PFC is engaged in effortful, controlled processing, which can lead to increased cognitive load and fatigue (43). This greater reliance on the PFC may not fully substitute for the more automatic and efficient processing of social information typically mediated by the FFA, amygdala, and STS. Consequently, while individuals with ASD may develop strategies to compensate for their face recognition deficits, these strategies might be less efficient and more taxing, potentially impacting their overall cognitive and emotional well-being (42).

## 3 Neural connectivity and structural anomalies of facial recognition in ASD

The section will explore the functional and structural brain connectivity in relation to facial recognition deficits in individuals with ASD. First, we will focus on how impaired functional connectivity between key regions, such as the FG, amygdala, PFC, and STS, contributes to social cognitive challenges. This section will highlight the impact of these disrupted neural connections on emotional processing and facial perception. Following this, the anatomical differences in these same regions will be examined, with emphasis on how structural anomalies in white matter tracts further impair communication between brain regions. By integrating both functional and structural perspectives, this review provides a comprehensive understanding of the neural mechanisms underlying facial recognition deficits in ASD.

### 3.1 Functional brain connectivity in ASD

In individuals with ASD, impaired functional connectivity between key brain regions involved in facial recognition contributes to social cognitive deficits. Vuilleumier et al. (44) demonstrated that amygdala damage correlates with underactivation of the FFA during facial recognition, while Anderson and Phelps (45) found that atypical amygdala activity affects the FFA’s ability to perceive facial emotions. Haxby et al. (30) further showed diminished connectivity between the FFA and other facial recognition regions in individuals with ASD, particularly in those with greater social deficits. Kleinhans et al. (46) similarly reported weaker connections between the FFA and amygdala, highlighting the importance of interaction between these regions for effective facial recognition.

Disrupted connectivity also involves the PFC and STS. Kana et al. (47) found reduced functional connectivity between the medial prefrontal cortex (mPFC) and amygdala during emotion processing, complicating compensatory mechanisms in ASD. This impaired connectivity persists into adulthood, as noted by Ammons et al. (48), further affecting social functioning. The PFC, while sometimes compensatory, cannot fully replace the automatic facial recognition processes typically handled by regions like the fusiform gyrus and STS. The importance of the STS in social cognition is further emphasized by studies examining its connectivity with other brain regions. For instance, Lombardo et al. (40) found that atypical STS connectivity with the mPFC and the amygdala in individuals with ASD correlated with difficulties in understanding social hierarchies and emotions. A study by Saitovitch et al. (49) also found that STS hypoactivity and reduced functional connectivity with other social brain regions were consistent markers of social cognition impairments in ASD. These findings suggest that STS hypoactivity contributes to a broader network dysfunction that underlies social cognitive impairments in ASD.

Additionally, resting-state fMRI studies further illustrate widespread connectivity issues. Schipul et al. (50) reported decreased connectivity between the amygdala and PFC at rest in individuals with ASD, which may hinder emotional processing and higher-order decision-making. Similarly, von dem Hagen et al. (51) identified atypical connectivity between the FG and the posterior superior temporal sulcus (pSTS), suggesting that the impaired coordination between facial perception and gaze processing regions contributes to the facial recognition deficits seen in ASD. These findings emphasize the importance of examining both structural and functional connectivity to fully understand the neural underpinnings of social cognitive impairments in ASD.

### 3.2 Anatomical differences and neural connectivity in ASD

In addition to functional anomalies in facial recognition networks, individuals with ASD exhibit structural differences that further contribute to their social cognitive impairments. Voxel-based morphometry (VBM) studies have consistently revealed volumetric alterations in key brain areas involved in social cognition. Ecker et al. (52) conducted a comprehensive VBM analysis, finding increased gray matter volume in the fusiform gyrus (FG) and reduced volume in the amygdala in individuals with ASD. These volumetric changes in regions responsible for processing facial identity and emotions suggest that anatomical anomalies may underlie the functional deficits observed in ASD. Similarly, McAlonan et al. (53) reported decreased gray matter in the superior temporal sulcus (STS), a region critical for interpreting dynamic facial features such as gaze direction and emotional expressions. Such structural variations may be linked to the challenges individuals with ASD face in social interactions, including difficulties with facial recognition and understanding social cues.

Neural connectivity studies provide further insights into how these anatomical differences disrupt communication between brain regions. Diffusion tensor imaging (DTI) has revealed altered white matter connectivity in ASD, particularly in pathways essential for social cognition. Lange et al. (54) found reduced fractional anisotropy (FA) in the inferior longitudinal fasciculus (ILF) and the uncinate fasciculus (UF)—fiber tracts that connect the FG to the amygdala and PFC. These pathways are vital for integrating facial identity and emotional information, and disruptions in their structural connectivity may contribute to the impairments in facial processing seen in ASD. Other studies utilizing advanced neuroimaging techniques, such as tractography, have reinforced these findings, showing that anomalies in these white matter tracts are associated with the severity of social and emotional difficulties (55, 56).

Functional brain connectivity analyses, particularly using resting-state fMRI, have also highlighted reduced communication between key facial recognition regions. Kleinhans et al. (46) demonstrated weakened connectivity between the FFA and the amygdala in individuals with ASD. This diminished functional integration of identity and affective processing supports the hypothesis that disruptions in the amygdaloid-fusiform network are central to the facial recognition deficits observed in ASD. More recently, Tseng et al. (57) extended these findings by showing that anomalies of functional connectivity between the FFA and other regions of the social brain network, such as the PFC and STS, may contribute to the broader social impairments seen in ASD. These studies emphasize the importance of investigating both structural and functional connectivity to understand the full extent of neural disruptions in ASD.

## 4 Implication and future directions

### 4.1 Potential implications for individuals with ASD

The neural anomalies found in key regions responsible for facial recognition, such as the FG, amygdala, STS, and PFC, have significant implications for the daily lives and functioning of individuals with ASD (**Table 1**). Reducing in FG activation, for example, can lead to difficulties in recognizing familiar faces, which are crucial for social interaction and relationship-building. This can result in increased social anxiety, avoidance behaviors, and challenges in forming personal or professional relationships. Atypical amygdala activation, which is critical for interpreting emotions, often results in difficulties for individuals with ASD to accurately read emotional expressions like fear, happiness, or anger. These deficits could lead to inappropriate or delayed responses in social settings, further complicating their ability to engage meaningfully in conversations or social activities. Additionally, hypoactivity in the STS hinders the processing of social cues such as eye gaze, making it harder for individuals with ASD to gauge the intentions or emotions of others during face-to-face interactions. Compensatory activation in the PFC, while helpful in some cases, imposes a heavy cognitive load on individuals with ASD. Social interactions that should be automatic become cognitively demanding, leading to fatigue, frustration, and eventual social withdrawal. This reliance on conscious effort in processing social information can make social engagements less enjoyable and more stressful, potentially exacerbating feelings of isolation. In sum, these neural anomalies contribute to the overall social challenges individuals with ASD face in daily life, including difficulty forming and maintaining relationships, understanding social contexts, and successfully navigating social environments. Addressing these challenges through early interventions aimed at mitigating neural dysfunction could have profound effects on improving social outcomes for individuals with ASD.

**Table 1:** Neural correlates of facial recognition deficits across brain regions.

Brain Regions Involved	Associated Neural Impairments	Description of the Correlation	Facial Recognition Deficits
Fusiform Gyrus (FG)	Reduced activation and structural anomalies	Reduced activation in the FG is linked to impaired facial identity processing; anatomical anomalies, such as reduced cortical thickness, contribute to these deficits.	Difficulty in recognizing and identifying faces.
Amygdala	Hypoactivation, hypersensitivity, and structural volume reduction	Atypical amygdala activation affects the ability to process emotional expressions, with both hyper- and hypoactivation reported in response to social stimuli. Anatomical reductions in volume are also observed.	Difficulty interpreting emotional expressions like fear, happiness, and anger.
Superior Temporal Sulcus (STS)	Hypoactivity and disrupted connectivity	STS hypoactivity leads to impaired gaze processing and difficulty interpreting dynamic social cues such as eye movements. Connectivity with facial recognition regions is weakened.	Difficulty understanding gaze direction and social intentions.
Prefrontal Cortex (PFC)	Increased activation and disrupted functional connectivity	The PFC compensates for deficits in facial recognition regions, leading to increased cognitive load and less efficient processing. Disrupted connectivity with the amygdala and STS complicates emotional regulation and social cognition.	Increased cognitive effort and fatigue during social interactions, leading to social withdrawal.
Fusiform Gyrus & Amygdala	Disrupted functional and structural connectivity	Weakened connectivity between the FG and amygdala hinders the integration of facial identity and emotional processing, exacerbating social cognitive impairments.	Reduced holistic processing of facial identity and emotions.
Inferior Longitudinal Fasciculus (ILF) & Uncinate Fasciculus (UF)	Reduced fractional anisotropy (FA) in white matter tracts	Disruptions in white matter connectivity between the FG, amygdala, and PFC contribute to impaired facial and emotional processing.	Impaired integration of facial identity and emotional information.

### 4.2 The future directions

#### 4.2.1 longitudinal and multimodal studies on neural development with emphasis on neural differences

Future research should integrate longitudinal tracking of neural development in individuals with ASD from early childhood to adulthood, employing advanced neuroimaging techniques like fNIRS, fMRI, MEG, and EEG (58, 59). These multimodal approaches will help identify how brain structures and functions related to face processing change over time, highlighting both differences and strengths rather than just deficits. Longitudinal studies should aim to understand the natural developmental trajectory and stability of face processing abilities, identifying individual variations and neural patterns in a neutral sense. This approach will provide a balanced view of ASD brain development, including how critical periods for interventions might emerge to enhance social cognition, without solely focusing on therapeutic outcomes. Modalities like fNIRS offer naturalistic and portable measurements, while DTI and functional connectivity MRI (fcMRI) explore structural and functional networks, allowing for a comprehensive understanding of neural diversity in ASD.

#### 4.2.2 Exploring individual differences and personalized approaches through AI and neuroimaging

Considering the heterogeneity in ASD, future research should explore individual neural mechanisms underlying face recognition deficits using neuroimaging biomarkers and advanced analysis methods (60–63). Identifying distinct neural and genetic markers will pave the way for personalized treatment approaches tailored to each individual’s unique neural and behavioral profile. Techniques like fMRI, EEG, and fNIRS can profile neural characteristics, while machine learning (ML) and computer vision can enhance predictive models for personalized interventions. For instance, ML models trained on both behavioral and neural data could aid in the prediction of treatment outcomes, while robot-assisted interventions (64, 65) provide a more holistic and individualized therapy strategy, considering both neurodiversity and unique developmental trajectories.

#### 4.2.3 Integration of neuroimaging techniques with advanced analytical approaches and neurodiversity consideration

The fusion of neuroimaging techniques, including functional connectivity analyses and fNIRS, with computational modeling and AI holds significant promise for understanding the complex and diverse nature of ASD. Specifically, fNIRS offers the benefits of portability and naturalistic assessment (58), and can be used alongside EEG, MEG, and MRI to explore both task-specific activation and resting-state functional connectivity in ASD. Machine learning algorithms, such as convolutional neural networks (CNNs), Vision Transformer (ViT) models, and hybrid approaches (e.g., CNN with XGBoost or Random Forest), can be applied to neuroimaging data to uncover patterns related to face recognition deficits. Incorporating these technologies enables a nuanced understanding of the neural underpinnings of ASD (41, 66), focusing on individual differences and neural diversity, and aids in developing targeted interventions and diagnostic tools informed by data from diverse populations.

#### 4.2.4 Leveraging AI for holistic understanding and support in ASD

Machine learning and big data analytics present new opportunities for refining ASD diagnosis and support strategies (58). Automated analysis of facial recognition patterns, eye movements, and neural data allows for the creation of sophisticated predictive models. Integrating neural indicators, such as brain activation and connectivity patterns, can improve the precision of ASD classification beyond traditional behavioral assessments. fNIRS and MEG offer promising data sources for these AI models, allowing for real-time analysis and potentially earlier detection of ASD symptoms (59). As AI models advance, they may facilitate more effective, targeted supports that adapt to the changing needs of individuals with ASD over time, emphasizing personalized growth and optimizing therapy effectiveness in alignment with the neurodiversity model.

## 5 Conclusion

This review highlights the complex neural basis of facial recognition deficits in individuals with ASD, emphasizing anatomical and functional anomalies across multiple brain regions. The FG, essential for facial identity recognition, shows reduced activation, with structural anomalies contributing to deficits. The amygdala, responsible for emotional expression processing, exhibits both hypoactivation and hypersensitivity, affecting facial emotion interpretation. The STS, involved in gaze processing and social cognition, shows hypoactivity, worsening social cue comprehension. The PFC, often compensatory, adds cognitive load, reducing facial recognition efficiency. Furthermore, the disruptions in structural and functional connectivity, such as between the FFA and amygdala, impair ASD’s facial identity and emotion integration. Neuroimaging studies (e.g., VBM, DTI) underscore the role of anatomical differences in these functional impairments, with disrupted connection between the PFC, amygdala, and STS suggesting inefficiency in compensatory mechanisms. Future research should use longitudinal, multimodal approaches to identify critical intervention periods. Personalized therapies, informed by neuroimaging and AI, hold promise for improving social cognition and quality of life in individuals with ASD through targeted interventions.
